# The System Is All Holes and No Cheese

**Published:** 2017-05-01

**Authors:** Krista Ramirez, Christopher Campen

**Affiliations:** Dr. Ramirez and Dr. Campen are pharmacists in the Greenville Health System.

## Abstract

A recent article in the *Chicago Tribune* revealed systematic failures among many retail pharmacies in detecting drug-drug interactions. This begs questions of what gaps exist in the current system of prescribing oral oncolytics and what advanced practitioners can do to keep patients safe.

An article recently published in the *Chicago Tribune* exposed significant systematic failures among many retail pharmacies in detecting drug-drug interactions, even when the prescriptions for the two interacting medications were presented at the same time (Roe, Long, & King, 2016). This issue leads to questions such as: What is happening with our oral oncolytics, which are significantly impacted by drug-drug interactions? How are we keeping our patients safe, from the point of prescribing to the time of drug administration and beyond? Where are the gaps in the system?

The *Chicago Tribune* article did a great investigative report on one small piece of a much bigger picture. Those of us who work in oncology as prescribers or in specialty-dispensing roles know the situation is much more complex than providing prescriptions for two medications that interact at the same time. In this editorial, we will use the "Swiss cheese model" of adverse drug outcomes to describe the current state of oral oncolytic prescribing and ways to improve the system (Horn & Hansten, 2004). The Swiss cheese model describes a layered scenario as a catch/stop for errors that may occur. Essentially, the more holes and/or the fewer layers of safety (cheese) in the system, the higher the risk for clinically impactful errors to potentially harm the patient.

## SLICE 1: PRESCRIBER KNOWLEDGE

Pharmacist knowledge of drug interactions is a gap noted by the *Chicago Tribune* article; however, with the increasing number of oral oncolytics approved by the US Food and Drug Administration (FDA), knowledge is also a gap that is likely widening among providers. Prescriptions for oral oncolytics are initiated by a physician in many oncology systems, although in some systems, advanced practitioners (APs) could initiate or be involved in renewing oral oncolytics. Prescribers must have general knowledge of common medications that can significantly affect drug levels of the substrate (i.e., oral oncolytics). In a prevalence study specifically on cancer patients, researchers found 58% of patients studied had at least one potential drug interaction, with 34% of interactions classified as major (van Leeuwen et al., 2011).

**Hole 1: Gap Analysis**

Prescribers should be provided educational updates that highlight drug interactions and have available, trained staff to help summarize key risks. All provider visits should include an assessment of new or changed medications. Patients should be reminded to bring all medications or a complete medication list to appointments. At the time of initiation of a new oral oncolytic, patients should be scheduled for a follow-up visit with an AP. This will allow for the time necessary (often at least 30 minutes) to conduct a sufficient review for drug-drug and drug-food interactions. The need for thorough medication reconciliation continues throughout treatment, with time set aside at follow-up visits to update medication lists and review changes with the same scrutiny.

## SLICE 2: COMPUTER SCREENING

Not all medications, including oral oncolytics, are entered into an electronic medical record (EMR) for drug interaction screening. Certain oral oncolytics must be faxed to an offsite specialty pharmacy, potentially bypassing the EMR. There is wide variability in how interactions are presented to the practitioner, if at all. In fact, there is no current standard of care addressing which interactions should be presented (McEvoy et al., 2017). In a comparison of accuracy and comprehensiveness among five drug-drug interaction software programs conducted by Kheshti, Aalipour, and Namazi (2016), none was found to be ideal.

**Hole 2: Gap Analysis**

All prescriptions should be reviewed for drug interactions at the point of prescribing, where specific degrees of severity can be reviewed. A focused effort should be placed on improvements among vendors who work with EMRs to provide drug interaction screening. Interaction screening should be designed to be consistent in approach to risk categorization and informative with study examples. For example, a patient taking a proton pump inhibitor with erlotinib (Tarceva) should be flagged as very high risk due to potential for reduced anticancer activity, and the EMR documentation should provide a brief paragraph on the study and associated decrease in systemic absorption/drug levels. Continuous improvement in tailored settings regarding which interaction warnings are presented to practitioners should be a goal at the organizational level. A "one-size-fits-all" approach to filtering interactions, even among specialties within the same organization, can lead to clinically significant interactions being filtered from view.

## SLICE 3: PHARMACIST KNOWLEDGE

A medication review by a specialized clinical pharmacist is invaluable when prescribing high-risk oral oncolytics. Patients are often required to use in-network pharmacies as determined by their insurance for specialty medications, which adds another complicating layer to an already risky prescribing practice. Patients may have medications at local retail pharmacies, mail-order retail pharmacies, and specialty pharmacies due to insurance restrictions, and this needlessly creates knowledge gaps regarding concomitant medications. Specialty pharmacies outside of the health system often fall short in delivering adequate medication list reviews and drug-drug interaction screens. It is not possible for a pharmacist to adequately review an oral oncolytic order without sufficient patient information.

**Hole 3: Gap Analysis**

Due to the ever-changing network requirements of insurance companies and the limited distribution of several oral oncolytics, there are a number of pharmacies that could potentially intercept a prescription—none of which have access to the EMR. All oral oncolytic medication orders should be reviewed for drug-drug interactions prior to release of the order from the EMR by an onsite oncology pharmacist. The pharmacist should have EMR access to ensure no details are overlooked, as medical history and concomitant disease states may provide clues to pharmacologic therapy not included on a medication list. In-clinic pharmacist review keeps the most critical elements of review right where the order originates, adding another layer of safety to the prescribing process. Pharmacists reviewing these orders should have extensive training in oral oncolytics, enabling them to quickly identify issues and provide expert pharmaceutical advice.

## SLICE 4: PATIENT EDUCATION

Patient education should be an ongoing process that continues beyond an initial chemotherapy education visit with the AP. Patients often develop new educational needs throughout treatment due to changing therapies for concomitant disease states or management of toxicities related to the oral oncolytic.

**Hole 4: Gap Analysis**

Potential drug-drug interactions should be discussed with the patient as part of the initial medication education with the AP. This initial education should be supplemented with new therapy counseling and routine follow-up evaluations by a specialty oncology pharmacist as part of a medication therapy management program. Following initial education, patients should be able to recognize potential dangers presented by medication use without oncology team awareness, and they should have a trained specialty pharmacist available to them at all times to verify that any new medication is both safe to use and documented in the EMR. When operating within the health system, specialty oncology pharmacists should actively contribute to the patient’s medical record to maintain an updated medication list for the entire oncology care team.

## CONCLUSIONS

We are concerned about a system full of holes. We recently had a patient with metastatic renal cell carcinoma present for clinical review in our health system specialty pharmacy. The patient had been on pazopanib (Votrient) for 6 months already, the initial prescription for which was e-prescribed directly to an outside pharmacy, bypassing the health system specialty pharmacy review process. We have a dedicated specialty oncology pharmacist who reviews all oral oncolytic prescriptions regardless of insurance pharmacy network restrictions prior to routing these prescriptions where the insurance requires them to be filled; however, this does require appropriate routing of the prescription at the time of prescribing.

The specialty oncology pharmacist conducted a detailed medication reconciliation, during which the patient confirmed that he takes omeprazole at 40 mg, which was already on the medication list, along with antacids when additional relief was needed. The drug-drug interaction warning with acid suppressants had been filtered from practitioner view based on organization-wide settings in an effort to reduce alert fatigue, and the outside specialty pharmacy had not asked him about his current medications, per his report. This patient had been receiving as much as 40% less active drug over the course of 6 months, and despite multiple safety checkpoints in place, no one identified the problem.

At costs potentially exceeding $10,000 a month for a drug alone, and at the risk of receiving subtherapeutic levels of a life-sustaining medication, we feel strongly that patients receiving oral oncolytics deserve better. The system is failing, and the problem only seems to be growing.
